# Case report: Durable response from tegafur/gimeracil/oteracil (S-1) combined with fruquintinib and sintilimab as a third-line treatment for MSS metastatic colorectal cancer with a BRAF V600E mutation

**DOI:** 10.3389/fonc.2024.1468532

**Published:** 2024-12-20

**Authors:** Chunxia He, Jiaxin Chi, Zhihua Du, Zhenjie Zhuang, Liuning Li

**Affiliations:** ^1^ Department of Medical Oncology, Guangdong Provincial Hospital of Chinese Medicine, The Second Clinical Medical College, University of Guangzhou Traditional Chinese Medicine, Guangzhou, China; ^2^ The Second Clinical Medical College, Guangzhou University of Chinese Medicine, Guangdong, Guangzhou, China; ^3^ Department of Pathology, Guangdong Provincial Hospital of Chinese Medicine, The Second Clinical Medical College, University of Guangzhou Traditional Chinese Medicine, Guangzhou, China

**Keywords:** microsatellite stable (MSS), metastatic colorectal cancer (mCRC), third-line therapy, tegafur/gimeracil/oteracil (S-1), fruquintinib, sintilimab, durable responses, case report

## Abstract

Patients with microsatellite stable (MSS) metastatic colorectal cancer (mCRC) who fail first- and second-line treatments face significant challenges in third-line therapy, where monotherapies often yield poor outcomes and limited survival benefits. The prognosis is particularly poor for mCRC with the unique molecular subtype of BRAF V600E mutation. This report describes sustained benefits from a third-line treatment regimen (SFS) combining tegafur/gimeracil/oteracil (S-1), fruquintinib, and sintilimab in a patient with BRAF V600E-mutated MSS mCRC. A 23-year-old woman was admitted with dizziness, and enhanced computed tomography (CT) and colonoscopy revealed colon cancer. Based on pathological and genetic testing, the final diagnosis was colon adenocarcinoma with lymph node and liver metastases (cT3N1M1, stage IVc, BRAF-V600E(+), MSS type). Following progressive disease (PD) after FOLFOX chemotherapy and surgery, the patient received 40 cycles of the SFS regimen (S-1 60 mg bid po d1–14 + fruquintinib 3 mg qd d1–21 + sintilimab 200 mg ivd q3w), achieving stable disease (SD). At the most recent follow-up, the patient has remained in sustained remission for over 3 years. The SFS regimen may be an attractive therapeutic strategy for patients with BRAF V600E-mutated MSS mCRC, warranting further evaluation in a larger patient cohort. We have registered a related clinical study (registration number: ChiCTR2300079188) and hope that the results will bring new hope for patients with MSS mCRC.

## Introduction

According to authoritative guidelines such as the National Comprehensive Cancer Network (NCCN), the standard third-line therapy for microsatellite stable (MSS) metastatic colorectal cancer (mCRC) with RAS and BRAF mutations includes regorafenib, fruquintinib, and TAS-102 (1). The clinical efficacy of standard third-line treatment is limited. Based on the CONCUR (2), CORRECT (3), China FRESCO (4), international RECOURSE (5), FRESCO-2, and Asia-Pacific TERRA studies (6), median progression-free survival (mPFS) ranged from 1.9 to 3.71 months, and median overall survival (mOS) ranged from 6.4 to 9.3 months, and these were worse with BRAF V600E mutation, with mPFS of only 1.8 months and mOS of only 4–6 months (7). More effective treatment options are needed.

Several small prospective studies (7–10) and retrospective studies have found that tegafur/gimeracil/oteracil (S-1) has shown some efficacy in the third-line treatment of mCRC. Preliminary results from several phase IB/II studies (11, 12) of fruquintinib combined with sintilimab for MSS mCRC have demonstrated good efficacy. Theoretically, fruquintinib can restore tumor cell sensitivity to chemotherapy through multi-target and multi-pathway mechanisms. Fruquintinib and chemotherapy can also improve the tumor microenvironment in refractory colorectal cancer patients, promoting the release of tumor-associated neoantigens and enhancing the effect of immune checkpoint inhibitors (ICIs).

The combination of immunotherapy, chemotherapy, and anti-angiogenesis therapy has shown encouraging anti-tumor activity in various refractory solid tumors. However, studies specifically supporting the use of the combination of S-1, fruquintinib, and sintilimab (SFS regimen) in the third-line treatment of MSS mCRC are lacking. The efficacy of the SFS regimen as a third-line treatment for MSS mCRC remains uncertain. Here, we report a case of MSS mCRC with BRAF V600E mutation who achieved a durable response after receiving the SFS regimen third-line treatment.

## Case report

**Figure 1:**
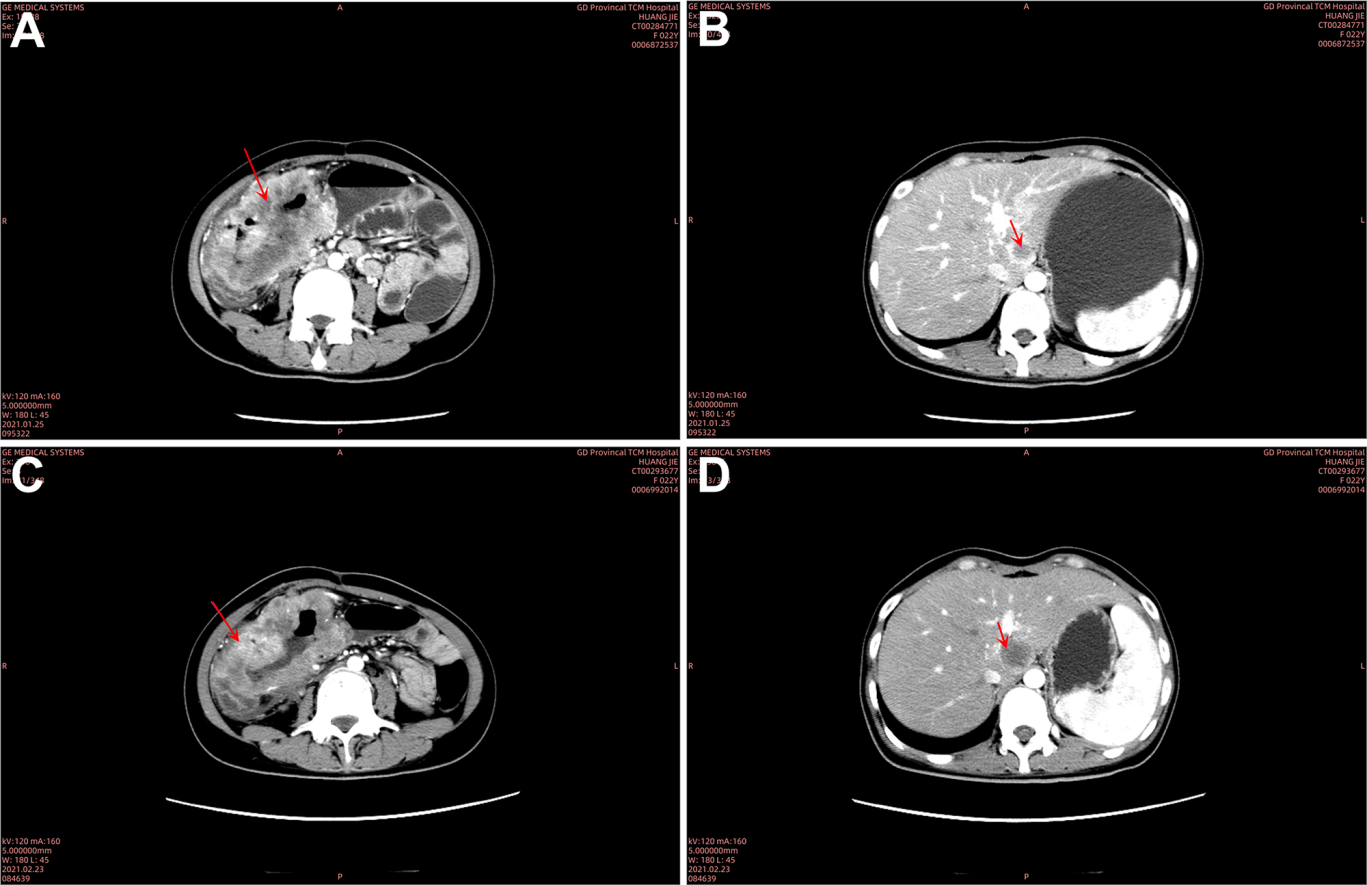
Chest and abdominal computed tomography (CT). January 25, 2021: **(A)** a large mass in the ascending colon, hepatic flexure, and transverse colon, highly suspicious for colon cancer. The mass extended beyond the serosa, infiltrating branches of the superior mesenteric artery, the greater omentum, and mesentery. Multiple enlarged lymph nodes (9.5 × 7.8 × 10.0 cm) were observed around the intestines, mesentery, and omental regions, raising suspicion for lymph node metastases. **(B)** A nodule at the S1–S2/3 junction of the liver (2.1 × 1.8 cm), suspected to be a liver metastasis. February 23, 2021: **(C)** mass in the ascending colon, hepatic flexure, and transverse colon remained, still suspected to be colon cancer. The tumor continued to infiltrate beyond the serosa and involved nearby vasculature and mesenteric tissues. Enlarged lymph nodes (9.8 × 7.8 × 10.0 cm) were similar to the findings from January 25, 2021, suggesting ongoing lymph node metastases. **(D)** The nodule at the S1–S2/3 junction of the liver had enlarged slightly (2.5 × 2.8 cm), consistent with the progression of suspected liver metastasis.

**Figure 2:**
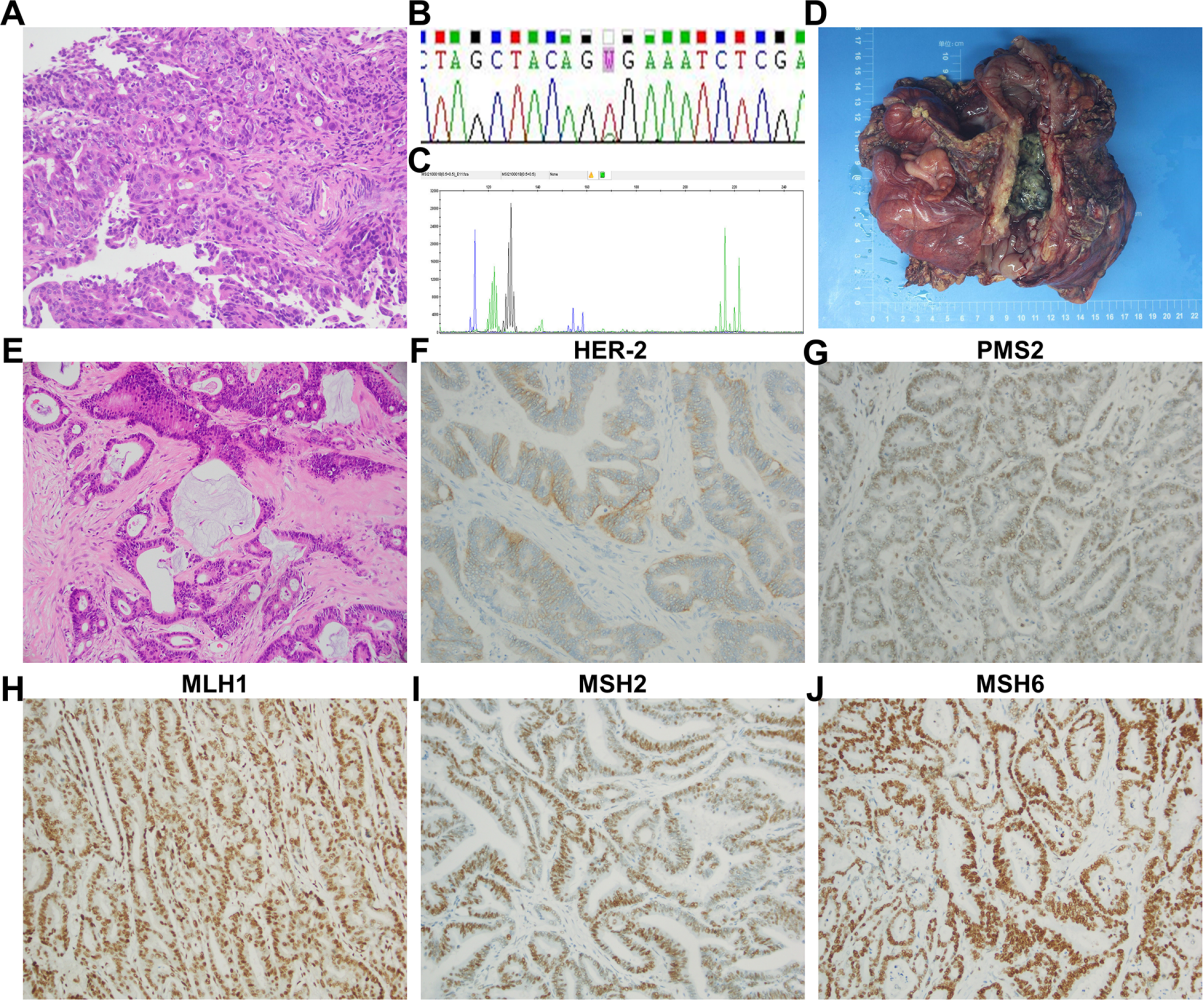
Colonoscopy and postoperative pathology. **(A)** Histopathological examination revealed adenocarcinoma of the transverse colon (hematoxylin and eosin, original magnification, ×200). **(B)** Sanger sequencing detected a BRAF c.1799T>A (p.V600E) missense mutation. **(C)** Microsatellite instability testing indicated the tumor was microsatellite stable (MSS type). Postoperative pathology: **(D)** the excised tumor measured 10.5 × 9.0 × 3.0 cm, ulcerated, and penetrating through the intestinal wall. **(E)** Histopathological analysis showed moderately to focally poorly differentiated adenocarcinoma, with partial mucinous differentiation (hematoxylin and eosin, original magnification, ×200). **(F–J)** Immunohistochemical staining showed HER2 negativity (score 1+), and no loss of expression was observed in MSH2, MSH6, MLH1, and PMS2 (original magnification, ×200).

A 23-year-old female patient presented with dizziness in January 2021 and was unable to walk on admission, with an Eastern Cooperative Oncology Group (ECOG) Performance Status (PS) score of 2. She had no significant past medical history or family history. Blood tests showed the following: hemoglobin (Hb), 53g/L; fecal occult blood test positive (3+); carcinoembryonic antigen (CEA), 50.49 ng/L; and carbohydrate antigen 19-9 (CA 19-9), 5,636.00 U/mL. Contrast-enhanced computed tomography (CT) indicated ascending colon cancer and multiple lymph node metastases (**Figure 1A**). A nodule at the S1–S2/3 junction of the liver was suspected to be a liver metastasis (**Figure 1B**). A colonoscopy showed colon cancer. Pathology showed adenocarcinoma of the transverse colon (**Figure 2A**). Immunohistochemistry results were as follows: MSH2, MSH6, MLH1, PMS2 (no loss of expression), CDX-2 (weakly +), CK(−), CK20(+), ER(−), PaX-8(−), and WT-1(−). Genetic testing indicated a BRAF c.1799T>A (p.V600E) missense mutation (**Figure 2B**), with no pathological mutations in KRAS, NRAS, and PIK3CA. No microsatellite instability was detected (MSS type) (**Figure 2C**). Based on these findings, her final diagnosis was adenocarcinoma of the colon with lymph node and liver metastases (cT3N1M1, stage IVc, BRAF-V600E(+), MSS type).

**Figure 3:**
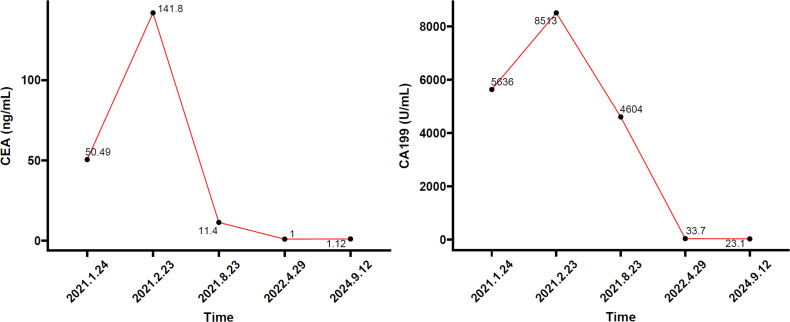
Tumor biomarker (CEA and CA 19-9 levels) change trend chart. CEA, carcinoembryonic antigen; CA 19-9, carbohydrate antigen 19-9.

After a red blood cell transfusion to improve anemia, the patient received one cycle of FOLFOX chemotherapy (oxaliplatin 85 mg/m^2^ + 5-FU 2.4 g/m^2^ q2w). Post-chemotherapy, she still experienced significant dizziness and fatigue, with Hb of 66 g/L, CEA of 141.80 ng/L, and CA 19-9 of 8,513.00 U/mL. **Figure 3** illustrates the temporal changes in the patient’s CEA and CA 19-9 levels, showing an increase in both markers following the initial treatment. On February 23, 2021, CT showed ascending colon cancer and multiple lymph node metastases similar to previous findings (**Figure 1C**). The metastatic tumor in the S1–S2/3 segment of the liver was slightly enlarged compared to previous findings (**Figure 1D**). The efficacy evaluation was progressive disease (PD).

**Figure 4:**
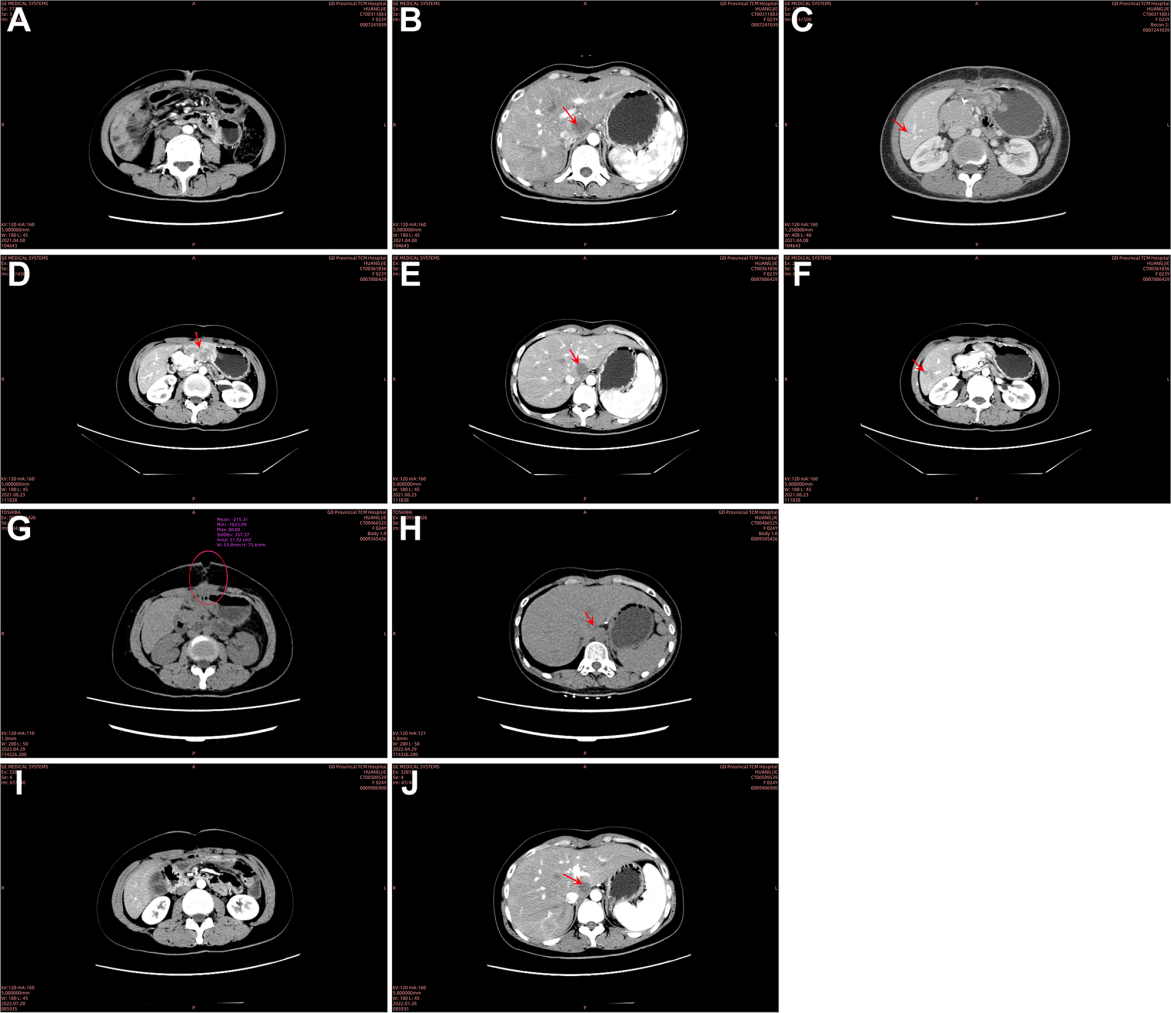
Follow-up computed tomography (CT) scans. April 8, 2021: **(A)** no residual mass was observed in the surgical area post-colon cancer resection. **(B)** The nodule at the S1–S2/3 junction of the liver (2.5 × 2.9 cm) persisted, consistent with suspected liver metastasis. **(C)** A new, slightly hypodense nodule (1.1 cm) was detected in the S6 segment of the liver, suspected to be a new metastatic lesion. August 23, 2021: **(D)** nodules and masses were identified in the gastric antrum, small omental sac, and hepatogastric space, suspicious for metastases (5.0 × 3.2 cm). **(E)** The nodule in the S1 segment of the liver (2.8 × 2.6 cm) remained similar to previous findings, consistent with liver metastasis. **(F)** The nodule in the S6 segment of the liver (1.0 cm) was also consistent with prior findings, suspected to be a liver metastasis. April 29, 2022: **(G)** the intestinal wall at the anastomosis site appeared unremarkable. Subcutaneous emphysema at the upper abdominal incision communicated with intra-abdominal air, forming an air-fluid level. Surrounding soft tissues were disorganized and poorly demarcated, raising suspicion for encapsulated effusion with infection and a potential entero-cutaneous fistula. **(H)** The nodule in the S1 segment of the liver (1.9 × 2.0 cm) persisted, consistent with a metastatic lesion. July 28, 2022: **(I)** the intestinal wall at the anastomosis site remained unchanged, and the subcutaneous emphysema at the original incision site had mostly resolved. Surrounding soft tissue swelling had decreased, and the anatomical structures were clearer. **(J)** The nodule in the S1 segment of the liver (1.9 × 2.0 cm) showed no significant changes compared to previous scans.

The patient failed standardized chemotherapy after one cycle, and severe anemia limited continued chemotherapy. The severe anemia was considered due to continuous bleeding from the colon cancer lesion. Given the patient’s very young age, lack of underlying diseases, a strong desire for treatment, and surgical opinion, she underwent palliative right hemicolectomy + partial gastrectomy + intraoperative microwave ablation of liver tumor (S1 segment metastasis) + adhesiolysis on March 1, 2021. The resected tumor measured 10.5 × 9.0 × 3.0 cm, ulcer-type, with invasion through the intestinal wall (**Figure 2D**). Postoperative pathology showed moderately to focally poorly differentiated adenocarcinoma and partially mucinous adenocarcinoma (approximately 40%) (**Figure 2E**). Immunohistochemistry results were as follows: P53 (70% +), Ki67 (80% +), HER2 (−, score 1 +) (**Figure 2F**), and no loss of expression was observed in MSH2, MSH6, MLH1, and PMS2 (**Figures 2G–J**). CT indicated that no clear mass was found in the surgical area (**Figure 4A**). The metastatic tumor in the S1–S2/3 segment of the liver was similar to previous findings (**Figure 4B**). A new metastatic tumor in the S6 segment of the liver was found (**Figure 4C**). From April 9, 2021, to August 5, 2021, she underwent seven cycles of FOLFOXIRI (irinotecan 165 mg/m^2^ + oxaliplatin 85 mg/m^2^ + 5-FU 2.4 g/m^2^ q2w) chemotherapy, with bevacizumab (5 mg/kg q2w) added from May 2, 2021, to August 5, 2021. In August 2021, CT showed nodules and masses in the gastric antrum above the anastomosis site, small omental sac, and hepatogastric space, suspected to be metastases (**Figure 4D**). The metastatic tumors in the S1 and S6 segments were similar to previous findings (**Figures 4E, F**). The efficacy evaluation was PD.

From August 2021 to April 2022, the patient received nine cycles of SFS regimen (S-1 60 mg bid po d1-14 + fruquintinib 3 mg qd d1–21 + sintilimab 200 mg ivd q3w). In April 2022, she developed a surgical site ulcer, and CT revealed subcutaneous and intra-abdominal pneumatosis, raising suspicion of an intestinal abdominal wall fistula (**Figure 4G**). The S1 liver metastasis had significantly decreased in size (**Figure 4H**), and the S6 lesion was no longer visible. The patient achieved a partial response (PR), but due to intestinal abdominal wall fistula and abdominal infection, the treatment was suspended. After debridement and infection control, the surgical wound healed well (**Supplementary Figure S1**). A repeat CT in July 2022 showed that the subcutaneous pneumatosis at the original upper abdominal incision had been basically absorbed (**Figure 4I**). The S1 segment metastases of the liver were the same as before (**Figure 4J**). From July 26, 2022, to the present, the patient continued to receive an SFS regimen (same as above) for a total of 40 courses. Subsequent regular follow-ups consistently demonstrated stable disease (SD). During this period, the levels of CEA and CA 19-9 exhibited a downward trend and stabilized (**Figure 3**).

The mCRC patient continued to progress after palliative surgery and medical treatment with first-line chemotherapy and second-line targeted combination chemotherapy. The patient achieved a good quality of life due to disease response in the third line of treatment with S-1 combined with fruquintinib and sintilimab. Overall, no serious adverse events were observed. The main adverse reactions were intestinal abdominal wall fistula, mild impairment of liver function, leukopenia, and alopecia. At the time of this report, the patient’s sustained response was ongoing for more than 3 years (**Supplementary Figure S2**).

## Discussion

The standard third-line therapy for MSS mCRC patients is regorafenib, fruquintinib, or TAS-102. However, existing studies indicate that the mOS and mPFS associated with these treatments are generally short, particularly in BRAF V600E-mutated mCRC patients, whose mPFS is only 1.8 months, highlighting the significant challenges of third-line therapy in this patient population. To our knowledge, this is the first report in which the S-1 combined with fruquintinib and sintilimab (SFS) regimen achieved both a durable response and good tolerability as a third-line treatment for MSS mCRC. Notably, this patient experienced a PFS exceeding 3 years following the failure of first- and second-line therapies.

In recent years, ICIs have been introduced in treating MSI-H/dMMR mCRC. However, approximately 85% of mCRC cases are immune-cold MSS tumors, which are unresponsive to ICI therapy. In China, the incidence of MSI-H in colorectal cancer patients is lower (7.7%–10.03%) (13), further limiting the efficacy of monotherapy with immune checkpoint inhibitors. Anti-vascular endothelial growth factor receptor-tyrosine kinase inhibitors (VEGFR-TKIs) can modulate the tumor immune microenvironment, supporting the theoretical basis for combining anti-VEGFR-TKIs and ICIs in mCRC. Several studies using anti-VEGFR-TKIs and ICIs have shown promising outcomes. The REGONIVO study (14) first demonstrated that the regorafenib and nivolumab combination regimen was significantly more effective than either agent alone in treating MSS mCRC, achieving an objective response rate (ORR) of 36% and an mPFS of 7.9 months. Similarly, Sun et al. (12) reported an mPFS of 6.4 months in the fruquintinib plus PD-1 inhibitor (FP) group compared to 3.9 months in the regorafenib plus PD-1 inhibitor (RP) group in refractory MSS mCRC. Preliminary findings from a phase IB/II study of fruquintinib combined with sintilimab for MSS mCRC in China (15) demonstrated an mPFS of 5.7 months and a mOS of 11.8 months. These findings suggest that fruquintinib plus PD-1 inhibitor may outperform regorafenib plus PD-1 inhibitor, supporting our choice of the fruquintinib and sintilimab combination for this patient.

Third-line chemotherapy alone in mCRC has limited benefit, with a mOS of 6–8 months (16). Meanwhile, a study by Lin et al. (17) found that more than half of Asian long-term survivors of mCRC still experience unmet needs, particularly among younger patients (<65 years), who often face financial burdens and limited treatment options during their therapeutic journey. This further underscores the importance of considering a comprehensive approach to treatment strategies in MSS mCRC patients, taking into account their age, financial status, and overall health condition. Anti-VEGFR-TKIs may enhance chemotherapy sensitivity by restoring cellular response mechanisms and overcoming multidrug resistance through multi-target, multi-pathway action, laying a foundation for potential synergy between TKIs and chemotherapy in mCRC. The SUNLIGHT study (18) compared TAS-102 plus bevacizumab (BEV) to TAS-102 alone in advanced CRC, with mPFS of 5.6 vs. 2.4 months [Hazard Ratio (HR) = 0.44] and mOS of 10.8 vs. 7.2 months (HR = 0.59). The Phase 2 study of Pfeiffer et al. (19) found that third-line BEV + TAS-102 significantly reduced the risk of death in BEV-naïve patients compared to those with prior BEV treatment, with HRs of 0.39 vs. 0.76. However, the retrospective study of Bang YH et al. (20) indicated that in patients who had previously been treated with BEV, the mOS with BEV combined with capecitabine was significantly worse compared to that of patients who had not received BEV treatment (p = 0.018). The retrospective study of Bang YH et al. (20) indicated that BEV combined with capecitabine significantly worsened mOS in patients with prior BEV exposure (p = 0.018). Hence, re-challenging BEV + chemotherapy as third-line treatment remains limited, and we did not choose the BEV + TAS-102 regimen for this patient.

In the treatment of CRC liver metastasis, a study by Sawano et al. (21) suggests that adjuvant chemotherapy should be considered for CRC patients following liver metastasis resection to reduce the risk of hepatic recurrence. Previous meta-analyses have demonstrated that, in the treatment of metastatic colorectal cancer, S-1 therapy offers comparable efficacy in PFS, ORR, and OS compared to those of 5-FU-based regimens, with a reduced toxicity profile (22). These findings support the use of S-1 in metastatic colorectal cancer patients who are intolerant to 5-FU-based therapies. Lee et al. (11) conducted a single-arm phase II study of 19 patients with mCRC who had failed standard oxaliplatin or irinotecan chemotherapy and were treated with S-1 with a median time to progression (TTP) of 2.1 months and a mOS of 11.3 months. This suggests that S-1, as a third-line treatment for patients with mCRC, can prolong the patient’s life and is safe and effective. For anti-VEGFR-TKI combined with S-1, Li et al. (23) conducted a single-arm phase II study to investigate S-1 combined with apatinib in the treatment of mCRC and included 30 patients with mCRC who had previously received more than two standard chemotherapies (irinotecan and oxaliplatin). The results showed that the mPFS and mOS were 7.9 months and 12.9 months, respectively. These results suggest that S-1 combined with anti-VEGFR-TKI may be used as a therapeutic exploration mode for the treatment of mCRC in the future, so we tentatively selected S-1 for the chemotherapeutic drugs of this patient.

The BRAF V600E mutation, linked with aggressive disease and poor response to chemotherapy, presents a significant clinical challenge. The optimal regimen for these cases—FOLFOXIRI combined with BEV post-palliative surgery—offers limited benefit, with PFS of approximately 4 months. Standard chemotherapy in BRAF V600E-mutated mCRC yields an ORR below 10%, mPFS of ~1.8 months, and mOS of 4–6 months (7). Vemurafenib, a BRAF V600E inhibitor, has shown efficacy in melanoma and lung cancer, but not in mCRC. The BEACON study (24) showed that combining encorafenib, binimetinib, and cetuximab achieved an mPFS of 8 months and mOS of 15.3 months, yet the lack of approval and high costs in China made this approach unfeasible for our patient.

Combining anti-VEGFR-TKIs, ICIs, and chemotherapy may enhance immune recognition and T-cell reactivity through immune microenvironment modulation, synergizing the efficacy of immune therapies (25). This combined strategy has been effective and safe in treating several refractory solid tumors, such as non-small cell lung cancer post-EGFR-TKI resistance, extensive-stage small cell lung cancer, and advanced cervical cancer [e.g., IMpower151, ORIENT31 (26), ETER701 (27), HARMONi-A (28), and BEATcc (29)]. Therefore, these results provide a basis for our patients receiving chemotherapy combined with anti-VEGFR-TKI and PD-1/PD-L1 combination mode. In our report, this patient sustained remission for more than 3 years. Therefore, it is promising to explore chemotherapy combined with ICIs and anti-VEGFR-TKI as a third-line treatment for mCRC.

Finally, the patient tolerated the adverse reactions well except for intestinal abdominal wall fistula and abdominal infection. However, we did not consider these adverse events to be related to this third-line combination regimen, as the patient received palliative surgery followed by continuous BEV treatment, considering the adverse reactions of BEV resulting in infection and abdominal wall fistula caused by poor wound healing. We stopped the drug for 3 months and then continued to use this regimen for nearly 3 years without related adverse reactions, and the fistula healed well.

## Conclusion

In this report, we describe a patient with MSS mCRC with BRAF V600E mutation who showed a durable response to third-line treatment with an SFS regimen. This strategy of SFS may provide effective third-line treatment options for patients with mCRC. Therefore, it is necessary to evaluate in future clinical trials (we have registered a Phase Ib single-arm study for further evaluation under clinical registration number ChiCTR2300079188).

## Data Availability

The datasets presented in this study can be found in online repositories. The names of the repository/repositories and accession number(s) can be found in the article/**Supplementary Material**.
