# Supplementary material for: Case report: Durable response from tegafur/gimeracil/oteracil (S-1) combined with fruquintinib and sintilimab as a third-line treatment for MSS metastatic colorectal cancer with a BRAF V600E mutation
Source: Front Oncol. 2024 Dec 20;14:1468532. doi: 10.3389/fonc.2024.1468532 (PMC11695214; doi:10.3389/fonc.2024.1468532)
Supplement: Supplementary file 1 [file Image1.pdf]

Supplementary Material

Supplementary Figures

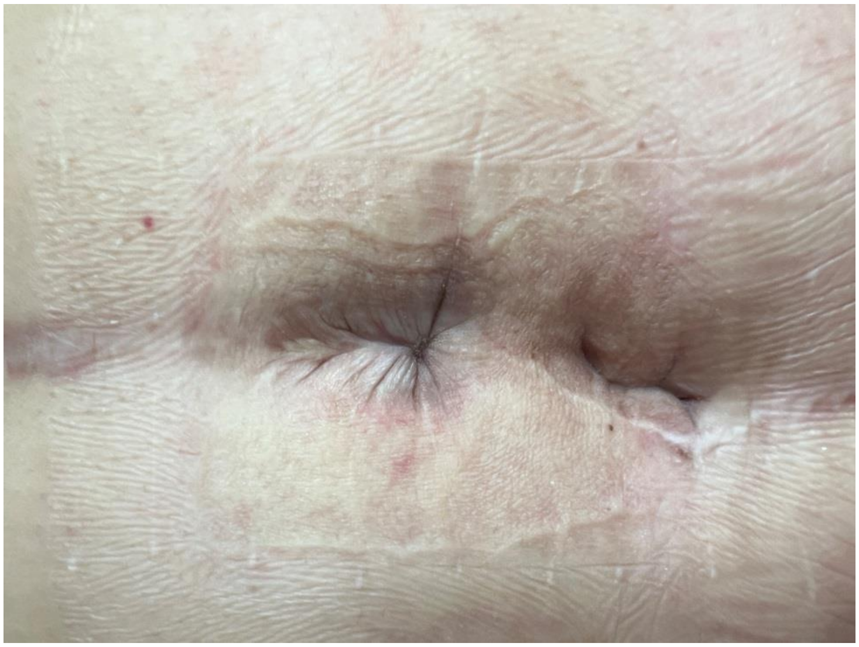

Supplementary Figure 1. Post-debridement wound healing

After debridement, dressing changes, and anti-infective treatment, the surgical wound healed satisfactorily.

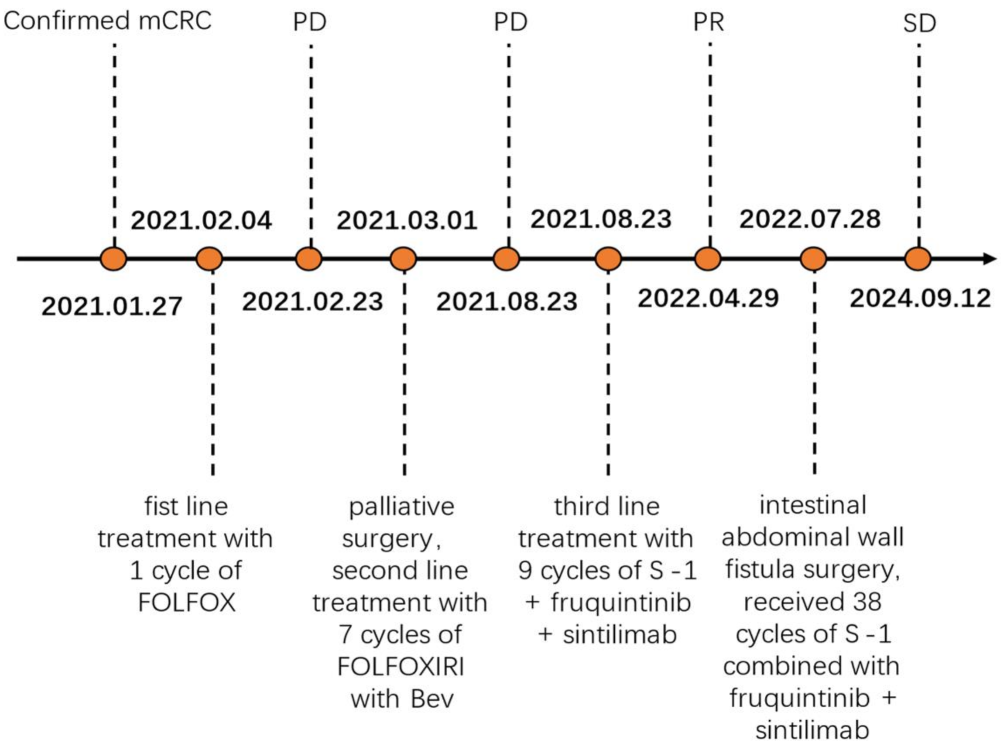

Supplementary Figure 2. Schematic picture showing the timeline of treatment procedure.
